# Microglial immunophenotype in dementia with Alzheimer’s pathology

**DOI:** 10.1186/s12974-016-0601-z

**Published:** 2016-06-02

**Authors:** Thais Minett, John Classey, Fiona E. Matthews, Marie Fahrenhold, Mariko Taga, Carol Brayne, Paul G. Ince, James A. R. Nicoll, Delphine Boche, MRC CFAS

**Affiliations:** Institute of Public Health, Department of Public Health and Primary Care, University of Cambridge, Cambridge, CB1 8RN UK; Department of Radiology, University of Cambridge, Cambridge, CB2 0QQ UK; Clinical Neurosciences, Clinical and Experimental Sciences Academic Unit, Faculty of Medicine, Southampton General Hospital, University of Southampton, Southampton, SO16 6YD UK; MRC Biostatistics Unit, Cambridge Institute of Public Health, Cambridge, CB2 0SR UK; Sheffield Institute for Translational Neuroscience, Sheffield University, Sheffield, S10 2HQ UK; Department of Cellular Pathology, University Hospital Southampton NHS Foundation Trust, Southampton, Southampton, SO16 6YD UK

**Keywords:** Microglia, Dementia, Alzheimer’s disease, Apolipoprotein E, Neuropathology

## Abstract

**Background:**

Genetic risk factors for Alzheimer’s disease imply that inflammation plays a causal role in development of the disease. Experimental studies suggest that microglia, as the brain macrophages, have diverse functions, with their main role in health being to survey the brain parenchyma through highly motile processes.

**Methods:**

Using the Medical Research Council Cognitive Function and Ageing Studies resources, we have immunophenotyped microglia to investigate their role in dementia with Alzheimer’s pathology. Cerebral cortex obtained at *post*-*mortem* from 299 participants was analysed by immunohistochemistry for cluster of differentiation (CD)68 (phagocytosis), human leukocyte antigen (HLA)-DR (antigen-presenting function), ionized calcium-binding adaptor molecule (Iba1) (microglial motility), macrophage scavenger receptor (MSR)-A (plaque-related phagocytosis) and CD64 (immunoglobulin Fcγ receptor I).

**Results:**

The presence of dementia was associated positively with CD68 (*P* < 0.001), MSR-A (*P* = 0.010) and CD64 (*P* = 0.007) and negatively with Iba1 (*P* < 0.001). Among participants without dementia, the cognitive function according to the Mini-Mental State Examination was associated positively with Iba1 (*P* < 0.001) and negatively with CD68 (*P* = 0.033), and in participants with dementia and Alzheimer’s pathology, positively with all microglial markers except Iba1. Overall, in participants without dementia, the relationship with Alzheimer’s pathology was negative or not significant, and positive in participants with dementia and Alzheimer’s pathology. Apolipoprotein E (*APOE*) ε2 allele was associated with expression of Iba1 (*P* = 0.001) and MSR-A (*P* < 0.001) and *APOE* ε4 with CD68, HLA-DR and CD64 (*P* < 0.001).

**Conclusions:**

Our findings raise the possibility that in dementia with Alzheimer’s pathology, microglia lose motility (Iba-1) necessary to support neurons. Conversely, other microglial proteins (CD68, MSR-A), the role of which is clearance of damaged cellular material, are positively associated with Alzheimer’s pathology and impaired cognitive function. In addition, our data imply that microglia may respond differently to Aβ and tau in participants with and without dementia so that the microglial activity could potentially influence the likelihood of developing dementia, as supported by genetic studies, highlighting the complexity and diversity of microglial responses.

## Background

Genome-wide association studies have implicated several inflammation-related genes as risk factors for Alzheimer’s disease, particularly in relation to innate immunity, suggesting a component of microglial activity is likely to be causal in the pathogenetic pathway [1]. The genetic studies also re-emphasized apolipoprotein E (*APOE*) genotype as the main risk factor for sporadic Alzheimer’s disease [1].

Microglia are the resident tissue macrophages of the central nervous system and thus have a key role in the immune surveillance of the brain [2]. They are normally highly motile cells with numerous long processes through which they are constantly sensing the brain environment for change [3]. Therefore, microglia react to any brain pathology including neuronal and synaptic damage and abnormal accumulations of proteins, fundamental features of Alzheimer’s disease [2]. Since the 1990s, it has been proposed that inflammatory processes may play an important role in the pathogenesis of Alzheimer’s disease. Early proponents of this idea suggested that neurotoxic substances (e.g. cytokines, complement) produced by microglia are an important cause of neuronal damage, which then provokes further microglial activation resulting in a self-perpetuating positive feedback loop [4, 5]. In addition, ageing, the main risk factor for Alzheimer’s disease, has been identified to be associated with a more pro-inflammatory/primed microglial state [6, 7].

Epidemiological retrospective studies also support the “inflammation hypothesis” of Alzheimer’s disease with evidence that non-steroidal anti-inflammatory drugs may be protective against the development of Alzheimer’s disease [8–10]. However, randomized controlled clinical trials of anti-inflammatory drugs in large cohorts of patients with established disease did not demonstrate benefit [11], perhaps reflecting our lack of knowledge of the specific roles of microglia at different stages in the development of Alzheimer’s disease.

The development of in vivo positron emission tomography (PET) imaging for microglia, using a ligand for translocator protein (TSPO), a protein present on the mitochondrial membrane and upregulated in neuroinflammation [12], has demonstrated microglial activation in Alzheimer’s disease [13]; although how the radioligand relates to the functional state of microglia is still unknown.

Considerable information is available about peripheral macrophages, to which microglia are related, which are highly plastic cells that adapt their behaviour to their environment undertaking different functions including recognition of pathogens, phagocytosis of microorganisms and cell debris, antigen presentation, cell toxicity and modulation of inflammation [2]. By extrapolation, microglia are likely to have a similar range of functions as supported by experimental models [14, 15] and *post*-*mortem* human studies [15, 16]. It is now recognized that cell morphology does not provide information on microglial function [2] and thus characterization of expression of microglial proteins related to different microglial functions (i.e. immunophenotyping) can offer a window into their functional status in a given situation.

**Fig. 1:**
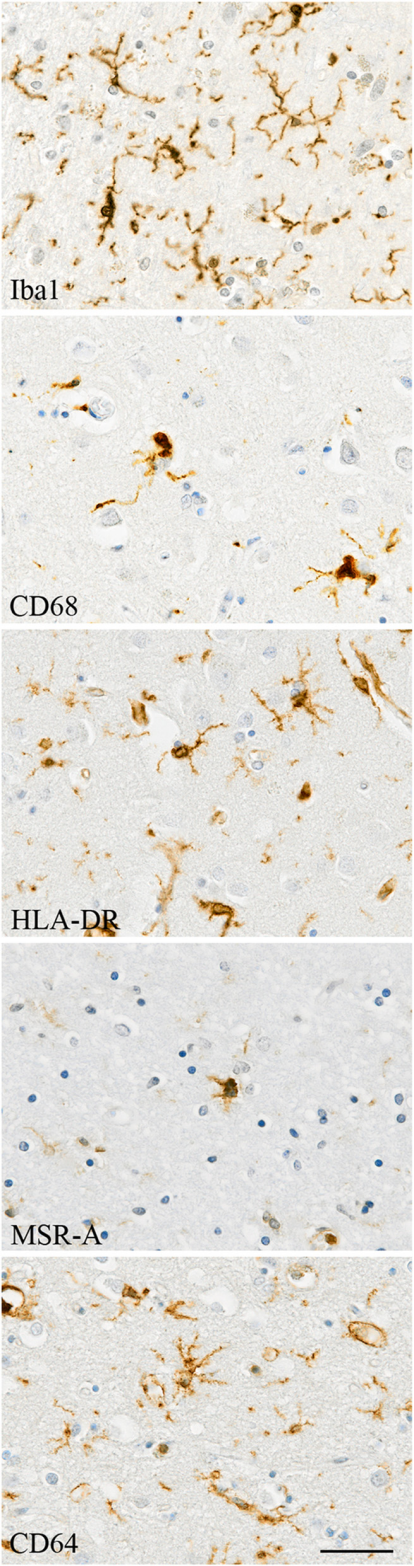
Illustration of microglia immunophenotyping in the human *post-mortem* brain using five antibodies related to different microglial functions. Haematoxylin counterstaining; *scale bar*: 30 μm

The Medical Research Council Cognitive Function and Ageing Study (MRC CFAS) is a multi-centred community-based study of the older population in the UK which includes participants with a full range of cognitive function from normal to those with dementia. The participants have been followed for over 20 years and offered the option for *post-mortem* brain donation. The result is a large cohort of cases which are unselected on the basis of cognitive function, dementia type and treatment and characterized in terms of clinical and neuropathological data [17, 18], allowing us to test our hypothesis on an unbiased representation of the elderly population. To test our hypothesis that different microglial functions are related to Alzheimer’s disease, we have immunophenotyped microglia in the CFAS cohort using antibodies to five proteins involved in different functions (Table 1, Fig. 1). This allowed us to assess whether a microglial immunophenotype is associated with (i) the presence of dementia, (ii) cognition, (iii) Alzheimer’s pathology and whether (iv) the effect of *APOE* genotype on the risk of dementia is related to the phenotype of microglia.

**Table 1:** Known functions of microglial proteins investigated

Proteins	Functions
Ionized calcium-binding adaptor molecule (Iba)1	Cytoplasmic protein constitutively expressed by microglia, upregulated in inflammation. Iba1 is involved in cytoskeletal reorganization, membrane ruffling of the microglial processes and actin cross-linking needed for cell migration [23], thus reflecting microglial motility and migration properties.
CD68	CD68 labels lysosomal and endosomal transmembrane glycoprotein of microglia, indicating phagocytic activity [33].
Human leukocyte antigen (HLA)-DR	HLA-DR is a Major Histocompatibility Class (MHC) II cell surface receptor which presents antigens to cells of the immune system eliciting an immune response, involved in the non-self recognition and upregulated in inflammation [34].
Macrophage scavenger receptor (MSR)-A	MSR-A is a lipoprotein receptor involved in direct ligand recognition and scavenging activity. Its mouse homolog, scavenger receptor A (SR-A), is associated with plaques and release of reactive oxygen species and neurotoxic substances by microglia upon stimulation with fibrillar Aβ [35]. We previously showed a clustering pattern of MSR-A-positive microglia round plaques in Alzheimer's disease [16] suggesting expression of MSR-A may cause immobilization of the microglia when they encounter plaques [16, 26].
CD64 (Fcγ receptor I)	CD64 is a cell surface receptor with high affinity for the Fc portion of immunoglobulin (IgG), triggering a monocyte/macrophage response [30]. Expression of CD64 reflects the presence of immunoglobulins in the brain and thus the involvement of systemic immunity [36]. Overall FcγRs are important for antibody-dependent cytotoxicity, antigen presentation via MHC, clearance of antibodies and phagocytosis [37].

## Methods

### The CFAS cohort

The CFAS study involves six centres in the UK (Liverpool, Cambridge, Gwynedd, Newcastle, Nottingham and Oxford). The design and methods have been described in detail elsewhere [17]. In brief, the project began in the early 1990s and recruited individuals living in the community aged 65 years and over. The main aims were to estimate the prevalence and incidence of cognitive decline and dementia, to determine the rate of progression of cognitive decline and survival and to identify risk factors for cognitive decline and dementia. Baseline prevalence screening of the cohort included sociodemographic, cognitive and physical health data. Participants were invited to consent to brain donation after death. The ascertainment of dementia status at death has been described in detail [18] and was based on review of information available from death certificates, last interview assessment and the informants’ information about participants’ function and cognition (Mini Mental State Examination (MMSE) score) during the last years of life. The brains of 299 participants were used in this study with the demographic and cognitive profile of the cohort described in Table 1. In 21 cases, insufficient information was available for a diagnosis of dementia to be made, and thus, these cases are excluded from the analysis.

### Assessment of Alzheimer’s pathology

We used the previous pathological evaluation of the CFAS cohort conducted by neuropathologists, blind to clinical data, using immunohistochemical or tinctorial methods [18]. The severity of diffuse plaques, neuritic plaques and tangles had been scored semi-quantitatively according to the Consortium to Establish a Registry for Alzheimer’s Disease (CERAD) protocol as either “none,” “mild,” “moderate” or “severe” [19]. For the analysis, as the score “severe” did not occur frequently, it was merged with “moderate”, and the score “mild” was merged with “none.” Cerebral amyloid angiopathy (CAA) was assessed in the meninges and parenchyma on a similar semi-quantitative scale. At the end of the assessment, a final neuropathological diagnosis of Alzheimer’s disease based on the distribution and severity of plaques and tangles but blind to any clinical information was made.

### Immunohistochemistry

The following primary antibodies were used: rabbit anti-human ionized calcium-binding adaptor molecule (Iba)-1 (Wako, Osaka Japan); mouse anti-human cluster of differentiation (CD)68 (clone PG-M1, Dako, Glostrup Denmark); mouse anti-human human leukocyte antigen (HLA)-DR (clone CR3-43, ThermoFisher Scientific, Loughborough UK); goat anti-human macrophage scavenger receptor (MSR)-A (R&D Systems, Abingdon UK); and mouse anti-human CD64 (immunoglobulin Fcγ-receptor I, R&D Systems, Abingdon UK) (Table 1, Fig. 1, Table 2).

**Table 2:** Antibodies and conditions

Microglial protein	Species	Clone/company	Dilution	Antigen retrieval technique
Iba1	Rabbit	Polyclonal/Wako	1:750	Pressure cooker citrate pH 6
CD68	Mouse	PG-M1/Dako	1:50	Microwave citrate pH 6
HLA-DR	Mouse	CR3/43/ThermoFisher Scientific	1:200	Microwave citrate pH 6
MSR-A	Goat	Polyclonal/R&D Systems	1:500	Microwave citrate pH 6
CD64	Goat	Polyclonal/R&D Systems	1:100	Microwave EDTA pH 8

Four micrometer sections of formalin-fixed paraffin-embedded tissue from the middle frontal gyrus, a region which is part of the CERAD neuropathology assessment for the diagnosis of Alzheimer’s disease, were used for immunostaining for microglial proteins. Immunohistochemistry was performed using the appropriate antigen retrieval methods for each primary antibody. Biotinylated secondary antibodies were from Dako (Glostrup, Denmark) and normal serum and avidin-biotin complex from Vector Laboratories (Peterborough, UK). Biotinylated antibody was visualized using the avidin-biotin-peroxidase complex method (Vectastain Elite ABC from Vector Laboratories (Peterborough, UK)) with 3,3′-diaminobenzidine (DAB, Vector Laboratories (Peterborough, UK)) as chromogen and 0.05 % hydrogen peroxide as substrate. All sections were counterstained with haematoxylin and then dehydrated before mounting in DePeX (VWR International, Lutterworth, UK). Cases were immunolabelled together in batches to ensure compatibility of staining, and sections incubated in the absence of the primary antibody were included as negative controls. For each antibody, a positive control was included to ensure staining consistency across the different batch runs.

### Quantification

Quantification was performed blind to the experimental group and identity of the cases. Images of the slides were taken starting from the sulcal depth adjacent to the middle frontal gyrus. For each antibody, 30 images of cortical grey matter at magnification ×20 were taken per case in a zigzag sequence along the cortical ribbon to ensure that all cortical layers were represented in the quantification in an unbiased manner. The acquired images were analysed using ImageJ (version 1.49 m, Wayne Rasband, NIH, USA), with a threshold applied to the image to select and measure the total amount of specific immunostaining. The same threshold setting was maintained for all images of all cases stained for the same antibody, and the area fraction of the measure function provided the proportion (%) of the stained area related to the total area of the image (expressed as protein load) [20]. A macro was designed to incorporate all the steps allowing automatic image processing and data collection. The data were then sent to the Department of Public Health and Primary Care for statistical analysis.

### Statistical analysis

The microglial data were analysed in relation to dementia status, cognition using the MMSE score [21] as a measurement of general cognition, specific pathological features of Alzheimer’s disease and *APOE* genotype. The relationships of Iba1, HLA-DR, CD68, CD64 and MSR-A expression with the different parameters were verified using weighted regression in which the 30 images acquired for each microglial protein were given the same 1/30 weight. Weighted logistic regressions were performed to verify the relationship between microglia and the dementia status; and weighted multiple linear regression analysis to assess whether microglial expression was related to cognition with adjustment for the gap between last interview and death. Weighted logistic regression analysis was used to assess the extent of the relationship between microglial expression and frontal lobe neurodegenerative pathologies. Participants with non-Alzheimer’s dementia were excluded. To verify the association of *APOE* genotype with microglial expression (dependent variables), weighted linear regressions were performed with ε2 and ε4 carrier status used as independent variables regardless of the number of alleles and with both alleles simultaneously present in the analysis. In addition, all analyses were adjusted for age of death and sex. All tests were two-tailed, and statistical analyses were performed using the statistical package STATA, version 12. A *P* value <0.05 was considered as significant.

## Results

### Characteristics of the cohort regarding dementia status

Among the 299 cases, 130 (47 %) cases did not have dementia at death. From the 148 participants who developed dementia, 83 (56 %) had plaques and tangles sufficient for the diagnosis of Alzheimer’s disease as the cause of dementia, and for 21 (7 %) cases, the dementia status was unknown. For the control group (participants without dementia), 66 (51 %) were women, the median age at death was 84 years (77–90) and the median MMSE score performed at the last assessment was of 25 (22–28). For the group with dementia, 102 (69 %) were women, including 64 % with Alzheimer’s pathology, with median age at death of 89 years (83–93). The median MMSE score performed at the last assessment for the participants with dementia and without Alzheimer pathology was 18 (11–23) and 11 (6–17) for people with dementia with Alzheimer’s pathology. Education was almost a constant, very similar across the groups and thus was not included as a covariate in our analysis (Table 3). Subsequent analysis omitted the group of dementia with non-Alzheimer’s pathology as these are a heterogeneous group and instead focused on comparisons between the participants with dementia and Alzheimer’s pathology and those without dementia.

**Table 3:** Characteristics of the cohort according to dementia status and microglial protein load (%)

	No dementia	Dementia with AD pathology	Dementia non-AD pathology	Unknown dementia status
	(*n* = 130)	(*n* = 83)	(*n* = 65)	(*n* = 21)
Number of women^a^	66 (51)	53 (64)	49 (75)	10 (48)
Age at death (years)^b^	84 (77; 90)	89 (83; 93)	89 (85; 93)	86 (84; 91)
Education (years)^b^	9 (9; 10)	9 (9; 10)	9 (9; 9)	9 (9; 9)
Years since last cognitive assessment^b^	1.1 (0.5; 1.8)	1.5 (0.8; 3.2)	1.7 (0.8; 3.0)	2.5 (2.0; 3.4)
MMSE at last assessment^b^	25 (22; 28)	11 (6; 17)	18 (11; 23)	25 (22; 27)
Iba1 load (%)^c^	2.346 (0.027)	2.047 (0.027)	1.824 (0.031)	2.372 (0.070)
CD68 load (%)^c^	0.090 (0.001)	0.100 (0.002)	0.088 (0.002)	0.054 (0.002)
HLA-DR load (%)^c^	0.213 (0.008)	0.277 (0.011)	0.143 (0.005)	0.101 (0.007)
MSR-A load (%)^c^	0.181 (0.003)	0.188 (0.003)	0.172 (0.003)	0.171 (0.005)
CD64 load (%)^c^	0.517 (0.006)	0.523 (0.007)	0.448 (0.007)	0.523 (0.013)

*AD* Alzheimer’s disease

^a^ *n* (%)

^b^Median (interquartile range)

^c^Linearized mean (linearized standard error)

### Microglia and cognition

Dementia status and a general cognitive function assessment (MMSE score) were used to assess cognition. The analyses were performed in relation to the microglial markers and included only participants without dementia and participants with dementia and Alzheimer’s pathology. Firstly, for dementia status (Table 4), there was a significant positive relationship with CD68 (*P* < 0.001), MSR-A (*P* = 0.010) and CD64 (*P* = 0.007) and a significant negative relationship with Iba1 (*P* < 0.001); no significant association was observed with HLA-DR. Thus, high loads of CD68, MSR-A and CD64 and a low Iba1 expression were related to the presence of dementia. Secondly, in relation to the MMSE score (Table 5), among the participants without dementia, there was a significant positive relationship with Iba1 (*P* < 0.001) and a negative relationship with CD68 (*P* = 0.033); no other significant association was observed. In the Alzheimer’s cohort, there was a significant positive relationship of MMSE score with CD64 (*P* = 0.023) and a negative relationship with CD68 (*P* < 0.001), MSR-A (*P* < 0.001) and HLA-DR (*P* < 0.001); no association was observed with Iba1. This indicates that poor cognition was related to higher expression of CD68, MSR-A and HLA-DR and lower expression of CD64. Overall, in both analyses (dementia and MMSE score), good cognition was associated with higher Iba1 and lower CD68 expression.

**Table 4:** Weighted logistic regression to analyse the relationship between microglial protein load (%) and dementia status in participants with and without Alzheimer’s dementia

Microglia (load (%))	OR	95 % CI (OR)	*P*
Iba1	*0.86*	(*0.82*; *0.89*)	<*0.001*
CD68	**3.55**	(**1.93**; **6.51**)	<**0.001**
HLA-DR	1.06	(0.96; 1.18)	0.250
MSR-A	**1.56**	(**1.11**; **2.19**)	**0.010**
CD64	**1.21**	(**1.05**; **1.39**)	**0.007**

These analyses only included participants without dementia or with dementia with Alzheimer’s pathology. Significant positive association (bold); significant negative association (italic)

**Table 5:** Weighted linear regression analyses investigating the relationship between microglial protein load (%) and the MMSE score in participants with and without dementia

Microglia (load (%))	No dementia			Dementia with Alzheimer’s pathology		
	*β*	95 % CI (*β*)	*P*	*β*	95 % CI (*β*)	*P*
Iba1	**0.37**	(**0.29**; **0.45**)	<**0.001**	−0.13	(−0.32; 0.05)	0.154
CD68	−*1.54*	(−*2.96*;−*0.13*)	*0.033*	−*12.17*	(−*15.13*; −*9.21*)	<*0.001*
HLA-DR	0.18	(−0.04; 0.41)	0.116	−*1.11*	(−*1.59*; −*0.64*)	<*0.001*
MSR-A	−0.69	(−1.38; 0.00)	0.051	−*4.94*	(−*6.82*; −*3.07*)	<*0.001*
CD64	0.27	(−0.03; 0.58)	0.076	**1.08**	(**0.15**; **2.02**)	**0.023**

These analyses only included participants without dementia or with dementia with Alzheimer’s pathology. Significant positive association (bold); significant negative association (italic)

### Microglia and Alzheimer’s neuropathology

Five neuropathological Alzheimer’s disease features previously assessed [18] were investigated in relationship with microglia: meningeal and parenchymal CAA, diffuse and neuritic plaques and tangles (Table 6 (A and B)). Among participants without dementia, the significant relationships observed between microglia and Alzheimer’s neuropathology were mainly negative, except for diffuse plaques which were positively related with four of the five microglial markers (Iba1, CD68, HLA-DR, CD64: *P* < 0.001) and Iba1 with neuritic plaques (*P* = 0.003) (Table 6A). The positive association between microglial markers and diffuse plaques regardless of Alzheimer’s disease is consistent with diffuse plaques being a relatively non-specific feature of ageing pathology [22]. In the participants with dementia and Alzheimer’s pathology, the significant relationships were mainly positive and stronger than those in the participants without dementia (Table 6B). Iba1 expression was significantly related to all neuropathological features. CD68 and MSR-A were strongly related with neuritic plaques (*P* < 0.001) and tangles (*P* < 0.001). HLA-DR and CD64 were significantly related to all neurodegenerative pathologies, except for parenchymal CAA and tangles (Table 6B).

**Table 6:** Weighted logistic regression analyses to investigate the relationship between microglia and Alzheimer’s pathology in participants with and without dementia

Microglia (load (%))	Meningeal CAA	Parenchymal CAA	Diffuse plaques	Neuritic plaques	Tangles
A. No dementia					
Iba1	0.92 (0.84; 1.01)	*0.72* (*0.65*; *0.79*)	**1.2** (**1.15**; **1.25**)	**1.07** (**1.02**; **1.13**)	*0.74* (*0.68*; *0.80*)
	0.064	<*0.001*	<**0.001**	**0.003**	<*0.001*
CD68	*0.09* (*0.01*; *0.99*)	*0.00* (*0.00*; *0.01*)	**8.62** (**3.83**; **19.40**)	*0.02* (*0.01*; *0.07*)	1.07 (0.06; 19.41)
	*0.049*	<*0.001*	<**0.001**	<*0.001*	0.965
HLA-DR	*0.27* (*0.16*; *0.47*)	0.79 (0.56; 1.12)	**2.24** (**1.77**; **2.83**)	1.02 (0.91; 1.15)	*0.07* (*0.02*; *0.25*)
	<*0.001*	0.183	<**0.001**	0.728	<*0.001*
MSR-A	*0.16* (*0.05*; *0.47*)	*0.03* (*0.00*; *0.35*)	1.42 (0.95; 2.13)	*0.24* (*0.14*; *0.40*)	0.44 (0.10; 1.96)
	*0.001*	*0.005*	0.09	<*0.001*	0.283
CD64	1.14 (0.77; 1.69)	*0.70* (*0.52*; *0.94*)	**1.86** (**1.57**; **2.20**)	*0.70* (*0.58*; *0.86*)	0.77 (0.45; 1.32)
	0.505	*0.018*	<**0.001**	*0.001*	0.343
B. Dementia with Alzheimer’s pathology					
Iba1	**1.36** (**1.28**; **1.45**)	**1.53** (**1.43**; **1.64**)	**1.37** (**1.24**; **1.51**)	**1.5** (**1.40**; **1.61**)	**1.14** (**1.07**; **1.21**)
	<**0.001**	<**0.001**	<**0.001**	<**0.001**	<**0.001**
CD68	1.07 (0.42; 2.73)	1.40 (0.43; 4.55)	*0.17* (*0.06*; *0.48*)	**31.65** (**11.54**; **86.81**)	**49.28** (**19.43**; **124.99**)
	0.892	0.576	*0.001*	<**0.001**	<**0.001**
HLA-DR	*0.62* (*0.52*; *0.74*)	1.07 (0.94; 1.22)	**3.22** (**2.50**; **4.15**)	**2.28** (**1.65**; **3.14**)	**1.74** (**1.42**; **2.13**)
	<*0.001*	0.302	<**0.001**	<**0.001**	<**0.001**
MSR-A	**2.43** (**1.26**; **4.66**)	**2.24** (**1.03**; **4.84**)	**2.44** (**1.15**; **5.21**)	**10.31** (**5.03**; **21.11**)	**3.24** (**1.74**; **6.04**)
	**0.008**	**0.041**	**0.021**	<**0.001**	<**0.001**
CD64	**2.15** (**1.71**; **2.71**)	**2.14** (**1.63**; **2.80**)	**21.59** (**14.22**; **32.78**)	**4.39** (**3.39**; **5.70**)	1.27 (0.99; 1.62)
	<**0.001**	<**0.001**	<**0.001**	<**0.001**	0.056

Values are presented as follows: OR (95 % CI (OR)), *P*. Significant positive association (bold); significant negative association (italic)

Interestingly, only one significant relationship was observed between cognition and the features of Alzheimer's pathology which was a negative association between tangles and MMSE score in the participants with dementia and Alzheimer’s pathology (Table 7).

**Table 7:** Linear regression analyses investigating the relationship between Alzheimer’s pathology and the MMSE score in participants with and without dementia

Alzheimer’s pathology	No dementia			Dementia with Alzheimer’s pathology		
	*β*	95 % CI (*β*)	*P*	*β*	95 % CI (*β*)	*P*
Meningeal CAA	0.48	(−2.87; 3.82)	0.779	0.49	(−3.25; 4.24)	0.794
Parenchymal CAA	−3.63	(−9.33; 2.07)	0.210	1.67	(−3.04; 6.39)	0.482
Diffuse plaques	0.74	(−0.67; 2.15)	0.299	0.50	(−3.45; 4.45)	0.800
Neuritic plaques	0.24	(−1.61; 2.09)	0.797	−2.43	(−5.84; 0.99)	0.161
Tangles	1.83	(−3.90; 7.56)	0.528	−*4.12*	(−*7.60*; −*0.65*)	*0.021*

These analyses only included participants without dementia or with dementia with Alzheimer’s pathology. Significant negative association (italic)

### Microglia and APOE genotype

We assessed the extent of the association of *APOE* genotype, the main genetic risk factor for sporadic Alzheimer’s disease, and altered microglial expression (Table 8). We detected that the possession of an *APOE* ε2 allele, known to be associated with reduced risk of Alzheimer’s disease, was significantly related to a high expression of Iba1 (*P* = 0.001) and MSR-A (*P* < 0.001) and a reduced amount of CD68 and HLA-DR (*P* < 0.001, respectively), whereas possession of an *APOE* ε4 allele, known to be associated with increased risk of Alzheimer’s disease, was significantly related to greater expression of CD68, HLA-DR and CD64, but a reduced amount of Iba1 (*P* < 0.001, respectively).

**Table 8:** Weighted linear regression analyses to investigate the association of *APOE* genotype with microglia

Microglia (load (%))	*β*	95 % CI (*β*)	*P*
ε2			
Iba1	**0.156**	(**0.068**; **0.244**)	**0.001**
CD68	−*0.010*	(−*0.014*; −*0.005*)	<*0.001*
HLA-DR	−*0.036*	(−*0.055*; −*0.017*)	<*0.001*
MSR-A	**0.017**	(**0.008**; **0.026**)	<**0.001**
CD64	0.020	(−0.001; 0.040)	0.060
ε4			
Iba1	−*0.166*	(−*0.237*; −*0.096*)	<*0.001*
CD68	**0.015**	(**0.011**; **0.019**)	<**0.001**
HLA-DR	**0.042**	(**0.019**; **0.065**)	<**0.001**
MSR-A	−0.004	(−0.010; 0.003)	0.317
CD64	**0.041**	(**0.024**; **0.058**)	<**0.001**

Significant positive association (bold); significant negative association (italic)

### Relationship between the different types of microglial markers

We explored the relationships between the microglial markers and found weak but significant relationships (*r* < 0.54, *P* < 0.015), except for CD68 and Iba1 which were not significantly related (*P* = 0.332; data not shown), supporting the hypothesis that microglial functions are performed relatively independently [16].

## Discussion

Our findings suggest that specific microglial proteins relating to diverse functions associate differently with cognition and features of Alzheimer’s disease pathology and that a change in microglial status may be important in the evolution of Alzheimer’s disease. We showed the association of Iba1 expression with the absence of dementia and scores of good cognition, whereas the presence of CD68, MSR-A and HLA-DR is related to dementia and scores of poor cognitive function. One of the main functions of microglia is to survey the brain parenchyma using highly motile cellular processes [3], which are regulated by actin polymerization and interaction with Iba1, an actin cross-linking protein crucial for actin bundling and microglial membrane ruffling [23]. Our finding raises the possibility that preserved microglial motility, being related to Iba1 expression, may protect against neurodegeneration putatively by facilitating active surveillance of the brain environment and rapid response towards any potentially neurotoxic insult. In contrast, the presence of HLA-DR (involved in antigen presentation and identified as a genetic risk factor for Alzheimer’s disease [1]) and phagocytic activity (CD68 and MSR-A) is detrimental to the brain, either by promoting or responding to neuronal damage. The absence of a significant relationship between cognition and Iba1 in the dementia cohort with Alzheimer’s pathology is consistent with the hypothesis that microglia may lose their motility potentially as a result of (i) AD-related microglial dysfunction due to senescence [24, 25], (ii) immobilization of microglia around plaques [16, 26] and/or (iii) an immunosuppressed status of microglia preventing them responding appropriately to the pathological environment [15, 24, 27]. Interestingly, the negative association of Iba1 with dementia status and yet its positive association with all five neuropathological features in established Alzheimer’s disease are seemingly contradictory findings that merit further exploration. These findings could be interpreted as consistent with microglial dysfunction and/or of the presence of an immunosuppressive environment inhibiting microglia from responding appropriately to the accumulated proteins.

Remarkably, the association between microglia and Alzheimer’s pathology appeared to change pattern between participants without and with dementia, with negative relationships with the different pathological features of AD prevailing in the absence of dementia and positive relationships in the dementia with Alzheimer’s pathology group. A key previous finding from CFAS neuropathology studies was that Alzheimer’s pathology is notably prevalent in elderly non-demented people [22], suggesting that additional factors over and above the plaques and tangles may be required to promote dementia. The results of the current study suggest that alterations in the microglial responses may, at least in part, provide that additional factor. More specifically, microglia seem to respond differently to Aβ and tau in participants with and without dementia, perhaps influencing the development of dementia rather than simply being the consequence of the ongoing neurodegeneration. In addition, the contention that motile microglia respond to pathology in a protective way is also supported by the finding that in participants without dementia, there is a negative relationship between tangles and the marker Iba1 (associated with absence of dementia and good cognition); and in the participants with dementia and Alzheimer’s pathology by a worse MMSE score related to tangles but not Iba1 expression. Our analysis of MMSE score and neuropathology confirmed that tangles are a better marker of cognitive impairment than Aβ plaques [28]. In the participants with dementia and Alzheimer’s pathology, the relationships between CD68, MSR-A and less strongly HLA-DR with tau pathology (i.e. tangles and neuritic plaques) are consistent with either microglial activity promoting or responding to tau accumulation. The association of CD68 with dementia, poor cognitive function and tau pathology (i.e. neuritic plaques and tangles) is particularly strong. CD68 is a protein present in phagocytic lysosomes within the microglia; however, it is not known whether microglia are causing harm by actively phagocytosing functioning neurons and synapses [29] or clearing up debris from damaged neurons and therefore simply responding to the neurodegeneration. CD64 expression is associated with the presence of dementia but not tangles. CD64 is the only high-affinity receptor for antibodies [30], reflecting the potential involvement of systemic immunity in the disease process [31]. For example, CD64 might participate in the immunosuppressed environment described in experimental and human studies of Alzheimer’s disease [15, 27] and thus to the impairment of microglial motility.

We demonstrated that *APOE* polymorphism may influence the microglia towards a protective (ε2 allele) or detrimental profile (ε4 allele), consistent with our clinical findings and previous studies [32]. The protective ε2 allele is associated with high expression of Iba1 (absence of dementia, good cognition), while the risk ε4 allele is associated with CD68, HLA-DR and CD64 (presence of dementia and bad cognition). However, microglia did not change the relationship between *APOE* genotype and dementia (analysis not shown), reinforcing *APOE* genotype as the stronger risk factor for Alzheimer’s disease.

## Conclusions

Despite the limitations inherent to any human *post-mortem* study, the major value of studying the human brain in this way is that it is a study of the disease itself rather than an experimental model of some aspect of the disease which does not inform specifically on human microglia. The novelty of our study resides in the combination of several microglial markers with known functions to investigate the role of microglia in Alzheimer’s disease in an unbiased population using a defined set of clinical and neuropathological parameters. Immunophenotyping microglia has demonstrated a weak relationship between the different microglial proteins studied revealing that (i) expression by microglia of one of the proteins does not necessarily predict the expression of the other proteins supporting the concept that microglia are able to adopt different functions relatively independently but also (ii) that different microglial populations may coexist within the brain as supported by the absence of association between CD68 and Iba1.

The complexity of microglial responses in the human brain as demonstrated in our study is important to reflect on, as this may explain the failure of anti-inflammatory agents in Alzheimer’s disease clinical trials and is likely to be a key to developing suitably tailored anti-inflammatory therapy to protect the ageing brain against neurodegeneration. Secondly, microglial activation can now be visualized and quantified in vivo with PET scans using specific ligands (e.g. TSPO). This technology is becoming widely used in different neurodegenerative diseases with an inflammatory component and in clinical trials to follow the effects of the drugs. Therefore, our findings highlight the importance of the phenotype expressed by microglia on the disease progression, an important parameter to consider when interpreting data from PET imaging for microglia, as one ligand is unlikely to reflect all aspects of microglial function [12, 13].

## Abbreviations

APOE, apolipoprotein E; CAA, cerebral amyloid angiopathy; CD, cluster of differentiation; CERAD, Consortium to Establish a Registry for Alzheimer’s Disease; CFAS, Cognitive Function and Ageing Studies; HLA, human leukocyte antigen; Iba1, ionized calcium-binding adaptor molecule; MMSE, Mini Mental State Examination; MRC, Medical Research Council; MSR-A, macrophage scavenger receptor-A; PET, positron emission tomography; TSPO, translocator protein; UK, United - Kingdom
